# Case Presentation: Pneumopericardium Following a Roux-en-Y Gastric Bypass

**DOI:** 10.51894/001c.166013

**Published:** 2026-07-31

**Authors:** Maya Shah, Lourd Al-Sharrak, Nolan Hayden

**Affiliations:** 1 College of Osteopathic Medicine Michigan State University

## 121

### Introduction

Pneumopericardium secondary to gastropericardial fistula is a rare but life-threatening complication following Roux-en-Y gastric bypass. One of the most serious associated complications is cardiac tamponade, which can rapidly become fatal if not recognized and managed promptly with a multidisciplinary approach.

### Case Presentation

A 73-year-old female with a history of rheumatoid arthritis, obstructive sleep apnea, chronic obstructive pulmonary disease, and Roux-en-Y gastric bypass complicated by recurrent gastrojejunal ulcers presented with worsening chest pressure. On presentation, imaging with CT of the chest demonstrated findings suspicious for a gastropericardial fistula originating from the gastrojejunal anastomosis, with gas and fluid extending between the pericardium and adjacent small bowel. Small-to-moderate bilateral pleural effusions with basilar atelectasis were also noted.

Further evaluation with chest radiography following oral Gastrografin administration demonstrated pneumopericardium with contrast traversing the gastroesophageal junction and extending into the pericardial space, confirming a gastropericardial fistula. CT esophagram supported these findings.

Interventional radiology performed pericardial drain placement and left-sided pigtail catheter insertion, with aspiration of approximately 200 mL of purulent fluid, gastric contents, and air from the pericardial space. The patient subsequently developed septic shock complicated by ischemic acute tubular necrosis and was started on broad-spectrum antibiotics. Thoracic surgery performed a pericardial washout with placement of a pericardial drain. Bariatric surgery subsequently performed laparoscopic takedown of the gastropericardial fistula with resection of the Roux limb, gastrogastrectomy, gastrostomy placement, and conversion to open surgery for removal of the Roux limb. The patient required vasopressor support in the surgical intensive care unit.

### Conclusion

Gastropericardial fistula is a rare but serious complication of Roux-en-Y gastric bypass that may present years after surgery. Prompt recognition on imaging and rapid multidisciplinary management are critical to prevent life-threatening complications including septic shock and cardiac tamponade.

